# Diversity among research coordinators in a pediatric emergency medicine research collaborative network

**DOI:** 10.1017/cts.2022.448

**Published:** 2022-08-22

**Authors:** Bashar S. Shihabuddin, Jessica Fritter, Angela M. Ellison, Andrea T. Cruz

**Affiliations:** 1 Division of Emergency Medicine, Nationwide Children’s Hospital, The Ohio State University College of Medicine, Columbus, OH, USA; 2 The Ohio State University College of Nursing, Columbus, OH, USA; 3 Abigail Wexner Research Institute, Nationwide Children’s Hospital, Columbus, OH, USA; 4 Division of Emergency Medicine, Children’s Hospital of Philadelphia, Philadelphia, PA, USA; 5 Division of Emergency Medicine, Children’s Tuberculosis Clinic, Texas Children’s Hospital, Baylor College of Medicine, Houston, TX, USA

**Keywords:** Diversity, research workforce, women in medicine, underrepresented minorities in medicine, research recruitment

## Abstract

We conducted a survey study of clinical research coordinators (CRCs) at the member institutions of the Pediatric Emergency Care Applied Research Network, to determine the demographic and linguistic characteristics of CRCs around the network, and any perceived impact of those characteristics on their duties. A total of 53/74 CRCs completed the survey. Most respondents identified as “female,” “white,” and “not Hispanic/Latino.” Most respondents felt that their race/ethnicity and their ability to speak a language other than English would positively impact recruitment. Four female respondents felt that their gender hindered their recruitment efforts and their sense of belonging within the research team.

## Introduction

Research team composition can enhance diversity among clinical research participants. Ensuring diversity in research subject recruitment increases the generalizability of research findings. In many institutions, research coordinators (CRCs) are responsible for critical elements in clinical research, including identifying, approaching, and consenting potential participants [1]. Recently, the association between diversity among research staff and patient recruitment has been described [2]. However, to our knowledge, there are no studies examining diversity among CRCs in clinical pediatric emergency medicine (PEM) research. Our primary objective was to describe the demographic characteristics of CRCs in a large PEM collaborative research network. Our secondary objective was to determine if CRCs perceived any impact of their demographic and linguistic characteristics on their duties.

## Methods

We conducted a 15-question electronic survey study of emergency department (ED) CRCs at member institutions of the Pediatric Emergency Care Applied Research Network (PECARN) between 4 January 2020 and 5 November 2020. PECARN includes 15 academic research pediatric hospitals and three emergency medical services research centers across 13 states, that involved 40 active research projects during the study period.

A waiver of written informed consent was granted for our study with Institutional Review Board approval. Contact information for the network CRCs was collated from the network personnel directory, which is updated on an annual basis. Four weeks after the initial electronic survey link distribution, a reminder message was sent to potential respondents. The survey included questions on demographics, linguistic abilities, education, and work experience, as well as the impact of race and ethnicity on patient recruitment and retention. Study data were collected and managed using REDCap® (Vanderbilt University; Nashville, TN). electronic data capture tools hosted at Nationwide Children’s Hospital. Descriptive statistics were used to analyze responses regarding demographics and proportion analysis was used to analyze binary response questions regarding perceived impact of demographic characteristics on CRC duties.

## Results

A total of 74 potential respondents were identified and received the electronic survey. A total of 53 complete responses (71.6%) were received and the responses are summarized in Table 1. Fourteen respondents (26.4%) identified as UIM (Black, Hispanic, or Latino). Twenty seven respondents felt that their race and/or ethnicity put patients and families from the same background at ease when approached for research studies (Table 2). On the other hand, 43 respondents felt that neither their gender nor ethnic background hindered their duties in any way (Table 2).

**Table 1.** Respondent self-reported demographics, work experience, and educational background

Characteristic/question	*N* (%)
Gender	
Male	12 (22.6)
Female	41(77.4)
Age range	
21–25 years	23 (43.3)
26–30 years	25 (47.1)
31–35 years	3 (5.6)
36–40 years	1 (1)
Greater than 40 years	2 (3)
Race*	
White	37 (64.9)
Black	3 (5.3)
Chinese	5 (8.8)
Asian Indian	4 (7)
Vietnamese	2 (3.5)
Korean	1 (1.7)
Arab/Middle Eastern	1 (1.7)
Other Asian	4 (7)
Hispanic/Latino*	
Hispanic-White	10 (18.9)
Hispanic-Other Asian	1 (1.9)
Non-Hispanic	42 (79.2)
Were you born in the United States?	
Yes	38 (71.7)
No	15 (28.3)
Highest degree achieved	
High school diploma	7 (13.2)
Associate/other certification or diploma	1 (1.8)
Bachelor	30 (56.6)
Master	11 (20.7)
Doctorate	5 (9.4)
Do you speak a language other than English?	
Yes	30 (56.6)
No	23 (43.4)
Is this your first experience with clinical research?	
Yes	28 (52.8)
No	25 (47.2)
Is this your first position in a research network?	
Yes	40 (75.5)
No	13 (24.5)

* Respondents were able to select more than one race/ethnicity.

**Table 2.** Respondents’ *perception of the impact of their background and characteristics*

Question	Yes (*N*)	No (*N*)	Unsure (*N*)
When approaching patients and parents for research studies, do you feel that your ethnic background places patient and parents *from your same background* at ease?	27	6	20
Do you feel that your gender or ethnic background hinders your job in any way?	4	43	6
Do you feel that your knowledge of the families or patients’ primary language (if their primary language is not English) makes you more successful in approaching those patients for participation in research studies?*	22	4	4

* Only answered by respondents who spoke another language as applicable.

Thirty respondents (56.6%) were knowledgeable in a language other than English, Spanish being the most common second language (Table 3). Significantly more respondents felt that their ability to speak the patient or family’s primary language, if that is other than English, made them more successful when approaching patients and families for research studies. Among respondents who spoke Spanish, 85% felt that their knowledge of the Spanish language made them more successful in approaching Spanish-speaking patients and families.

**Table 3.** Languages other than English reported as spoken by respondents, respondents were able to list more than one language

Language	*N*
Spanish	12
French	3
Hindi	3
Mandarin	3
Cantonese	2
Italian	2
Portuguese	2
Afrikaans	1
Arabic	1
Bosnian	1
Croatian	1
Serbian	1
Miña	1
Gujarati	1
Sinhalese	1
Korean	1
Mongolian	1
Punjabi	1
Telugu	1
Urdu	1

Four female respondents, all of whom identified as “white,” and three of whom identified as Hispanic, felt that their attributes hindered their responsibilities. As a follow-up to this question, respondents who felt that their background hindered their responsibilities were asked to describe their experiences. The descriptions by the respondents are listed below:

- *As a female, I sometimes feel as though I am treated differently than my male counterparts both in rooms and working within the research team.*
- *As a minority it is sometimes difficult to interact with some families.*
- *Because I am Hispanic and have a Hispanic last name but I do not speak Spanish I have to use an iPad interpreter. I feel that if I could speak Spanish I would identify better with that population.*
- *I believe my gender, as a female, hinders my job due to my experiences with my male counterparts. I've seen the differences in the way patients/families interact with male CRCs when they approach for consent vs. when I approach for consent. It seems to be that they trust my male counterparts more and are more willing to do studies with male CRCs.*

## Discussion

Our study illustrated the state of diversity among research personnel in a large, successful, pediatric emergency medicine research network. About 25% of respondents in our cohort were from UIM, lower than the UIM population of the United States, but higher than the number of UIM in the US healthcare workforce [3]. Diversity among research personnel can support cultural humility, a process of self-reflection among team members that provides open awareness of biases, privileges, and the limitations of one’s own knowledge [4]. This continuous process could enhance their capabilities in subject approach and recruitment, and complement any existing cultural competency training [5]. In turn, cultural humility would promote partnerships between the research team and research subjects which is mutually beneficial. Cultural humility can diversify recruitment efforts, decrease participant drop out, and encourage future research participation.

Respondents in our cohort who spoke a language other than English found that that ability positively impacted recruitment efforts. Our results support fostering language proficiency among research personnel to improve approach and recruitment in clinical research. Research personnel may be able to establish better rapport with patients when they share a common characteristic [6].

We were concerned about the experiences described by some female respondents. Despite constituting most of our cohort, some female respondents believed their gender made their responsibilities more difficult. Even further, those difficulties were not only related to recruitment efforts, but working within the research team. Identifying the incidence of such discrimination, and eliminating them, is essential to creating a safe and diverse work environment for all members of the research team. Further, research team leaders, managers, and investigators can use the principals of cultural humility to support their team makeup and ensure a continued sense of belonging and equity among team members. Cultural humility can augment current cultural competency efforts and can be used when assessing hiring processes, as well as on-boarding procedures and interval assessments of study procedures. Interventions, such as writing job descriptions for CRC position using the principles of cultural humility and allowing applicants to identify their strengths and potential contributions to clinical research freely, can be valuable in hiring and maintaining a diverse research team [7].

### Limitations

Our study had several limitations. The survey questions were not internally validated prior to their distribution, and we did not ask any specific questions related to experiences with discrimination or harassment. We felt that asking those questions in a de-identified survey would hinder any efforts to provide aid or care to victims of such discrimination. We also did not ask about proficiency in languages other than English; therefore, we could not determine whether language proficiency had any effect on patient and family approach and consent. The survey questions regarding respondents’ perceived impact of their characteristics on their responsibilities were open ended aimed at general attitude towards any effects on their duties. In future work, we will ask specifically about enrollment and requirement, as well as the work environment. Finally, we were unable to correlate our survey findings with any patient enrollment data as, typically, data on concordance or discordance between research personnel characteristics and patient characteristics is not readily available.

## Conclusions

Our study found that PECARN CRCs are from diverse backgrounds, but the percentage of CRCs who identify as UIM is lower than the percentage of UIM in the general population, though higher than the percentage of UIM in the US healthcare workforce. Some female CRCs in our cohort encountered difficulties in the course of their work based on their gender. Increasing cultural humility among research personnel and supporting women in research to ensure a safe and inclusive working environment should be prioritized by executive leadership.
